# Cholesterol Increases Lipid Binding Rate and Changes Binding Behavior of *Bacillus thuringiensis* Cytolytic Protein

**DOI:** 10.3390/ijms19123819

**Published:** 2018-11-30

**Authors:** Sudarat Tharad, Öykü Üzülmez, Boonhiang Promdonkoy, José L. Toca-Herrera

**Affiliations:** 1 Institute for Biophysics, Department of Nanobiotechnology, University of Natural Resources and Life Sciences (BOKU), 1190 Vienna, Austria; oeykue.uezuelmez@meduniwien.ac.at; 2 National Center for Genetic Engineering and Biotechnology (BIOTEC), National Science and Technology Development Agency (NSTDA), Pathumthani 12120, Thailand; boonhiang@biotec.or.th

**Keywords:** *Bacillus thuringiensis*, Cyt2Aa2 protein, cholesterol, QCM-D, AFM, lipid binding

## Abstract

Cytolytic protein (Cyt) is a member of insecticidal proteins produced by *Bacillus thuringiensis*. Cyt protein has activity against insect cells and mammalian cells, which differ in lipid and cholesterol composition. This study presents the lipid binding behavior of Cyt2Aa2 protein on model membranes containing different levels of cholesterol content by combining Quartz Crystal Microbalance with Dissipation (QCM-D) and Atomic Force Microscopy (AFM). QCM-D results revealed that cholesterol enhances the binding rate of Cyt2Aa2 protein onto lipid bilayers. In addition, the thicker lipid bilayer was observed for the highest cholesterol content. These results were confirmed by AFM. The analysis of protein surface coverage as a function of time showed a slower process for 5:0 and 5:0.2 (POPC:Chol) ratios than for 5:1 and 5:2 (POPC:Chol) ratios. Significantly, the Cyt2Aa2-lipid binding behavior and the protein–lipid layer were different for the 5:3 (POPC:Chol) ratio. Furthermore, AFM images revealed a transformation of Cyt2Aa2/lipid layer structure from strip pattern to ring shape structures (which showed a strong repulsion with AFM tip). In summary, cholesterol increases the binding rate and alters the lipid binding behavior of Cyt2Aa2 protein, although it is not required for Cyt2Aa2 protein binding onto lipid bilayers.

## 1. Introduction

*Bacillus thuringiensis* (*Bt*) is a soil Gramm-negative bacterium. *Bt* is wildly known because of its insecticidal property [1]. Insecticidal toxins are produced at different stages of cell growth. A vegetative protein (Vip) is initially produced at the vegetative phase, whereas crystal protein (Cry) and cytolytic protein (Cyt) are subsequently found at sporulation phase. Although the toxins have insecticidal properties, their amino acid sequences are dissimilar [2,3,4]. To improve the efficiency of insecticidal activity, it has been necessary to investigate lipid–protein binding mechanisms. 

Cyt protein shows in vitro activity against a variety of cell types: insect cells, mammalian cells, and bacteria [5,6]. For in vivo activity, Cyt protein exerts its activity especially against insect vectors such as mosquitoes and black flies [7,8]. Cyt protein is classified into three classes: Cyt1, Cyt2, and Cyt3, based on their amino acid sequences identity [9]. A single domain structure of β-sheet core sandwiched by α-helix is conserved among the Cyt protein family [3,10,11,12]. Unlike Cry toxin, a protein receptor is not required for a Cyt toxin to bind on a target cell. As shown in previous studies, the phospholipid and sphingomyelin containing unsaturated acyl chains are necessary for lipid membrane binding of Cyt toxin [13,14,15,16]. As mention above, Cyt toxin can disrupt many cell types whose lipid composition of cell membranes is different. 

Cholesterol is a fundamental component of the cell membrane, and its amount in lipid membranes varies among organisms and cell organelles [17]. The interaction between cholesterol and phospholipid molecules has been extensively studied in order to determine the importance of cholesterol on lipid raft formation [18]. In this work, we are interested in the importance of cholesterol in the interaction between Cyt2Aa2 (from *B. thuringiensis* subsp. *darmstadiensis*) and lipid bilayer, including the protein–lipid binding behavior and the final structure of the hybrid layer. The use of artificial lipid membranes as model systems (i.e., lipid vesicles, black lipid membranes, lipid monolayers, and supported lipid bilayers) reinforces current research on protein–lipid interactions, providing new evidence about the mechanism of the biological process involved. In addition, such model systems can be also utilized for biosensor technology [19]. Thus, supporting lipid bilayers (SLBs) are a suitable system to study Cyt protein–lipid interactions. In our investigation, the cholesterol content has been varied in the SLB model to mimic cell membranes that have shown activity in response to Cyt protein [5]. In particular, the lipid bilayer models were built with phosphatidylcholine (POPC), mono-unsaturated lipids, and cholesterol by mixing a fixed amount of POPC with different levels of cholesterol content. In this system, the lower ratio of POPC:Chol (5:0 and 5:0.2) corresponds to the insect cell membrane [20], while the higher content of cholesterol (5:1, 5:2, and 5:3) can be used as a model system for mammalian cells [17,21]. 

To elucidate the lipid binding behavior of Cyt2Aa2 protein and the nano-structure of the final protein–lipid bilayer system, quartz crystal microbalance with dissipation (QCM-D) was combined with atomic force microscopy (AFM). In this study, we report that cholesterol promotes the lipid binding rate and changes the lipid binding behavior of Cyt2Aa2 protein at the highest cholesterol content.

## 2. Results

### 2.1. Changes in Lipid Bilayer Properties Upon Increasing of Cholesterol

**Figure 1:**
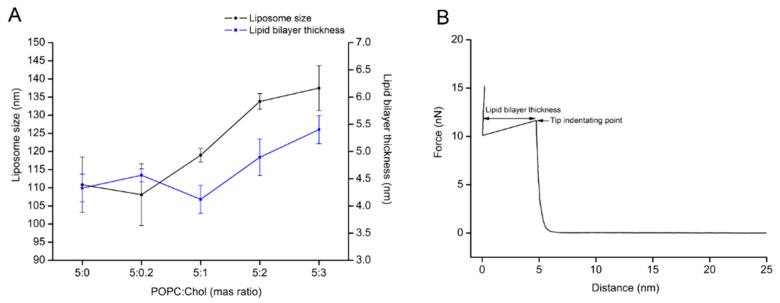
Determination of the liposome size and the lipid bilayer thickness as a function of different levels of cholesterol content in the lipid membrane. (**A**) The liposomes were determined size by dynamic light scattering. The lipid bilayers were formed by liposome fusion method. After the topographic images were collected, then tip indentation experiments were carried out to determine the lipid bilayer thickness (data were collected from 40 points over the surface image of lipid bilayer). (**B**) The lipid bilayer thickness was measured from the force–distance curve.

To carry out protein–lipid interaction studies, supported lipid bilayers (SLBs) were formed by liposome fusion method. POPC/Chol liposomes of different weight ratios were tested as candidates for lipid bilayer formation by QCM-D and AFM. First, the liposome size and bilayer thickness of the lipid/Chol were evaluated. Figure 1A shows that the liposome size and lipid bilayer thickness of the lipid mixture from 5:1 to 5:3 (POPC:Chol) ratios tends to increase relative to the amount of cholesterol in the lipid bilayer. In addition, the zeta potential (ζ) of liposomes was measured, while the obtained values were similar, ranging from –3.0 to −4.0 mV (Table S1). Furthermore, QCM-D measurements showed that the changes in ∆F and ∆D values for lipid bilayer were almost constant, except for the lipid mixture 5:2 (POPC:Chol) ratio, which was slightly higher (see Table 1). Here, it is important to point out that bilayer formation was only a spontaneous process for the pure POPC liposome and the lowest cholesterol content in the liposome (5:0.2 ratio). The ∆F and ∆D plots for other POPC:Chol ratios indicated three steps: liposome adsorption, liposome fusion, and further liposome formation. However, lipid bilayers with higher cholesterol could be built by buffer rinsing after liposome adsorption and fusion. Additional decreasing of ∆F (increasing of ∆D) was observed ca. 10 min after the maximum liposome adsorption (the first peak of ∆F and ∆D). To obtain characteristic ∆F and ∆D values of a lipid bilayer after liposome fusion, further changes in the signal were reduced by buffer rinsing (Figure S1). Complementary AFM imaging and force–distance curves were carried out to confirm lipid bilayer formation. The results showed that POPC/Chol bilayers were formed for all ratios (Figure S2). Subsequently, the bilayer thickness was estimated by force–distance measurements (Figure 1B). The indentation of the bilayer delivered the following values: 4.3 ± 0.3 nm, 4.5 ± 0.1 nm, 4.1 ± 0.3 nm, 4.8 ± 0.3 nm, and 5.5 ± 0.3 nm for 5:0, 5:0.2, 5:1, 5:2, and 5:3 (POPC:Chol), respectively. The lipid bilayers were thicker for the lipid mixtures of 5:2 and 5:3 (POPC:Chol) compared to pure POPC lipid bilayer (the liposome size also followed the thickness trend, see Figure 1A).

### 2.2. Increasing of Protein Adsorption Rate and Lipid Binding Behavior as Function of Cholesterol Content 

**Figure 2:**
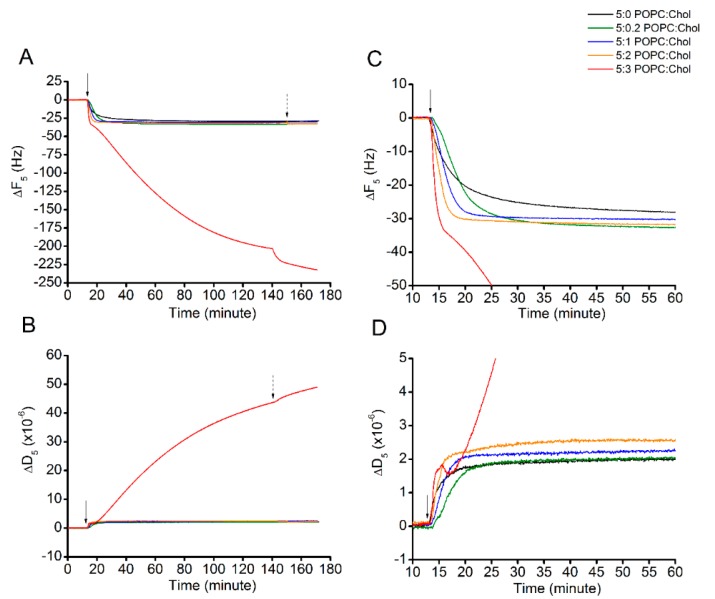
Protein binding on lipid bilayers with different levels of cholesterol content. The lipid bilayers were formed on top of the sensor surface via liposome fusion. This is represented as zero value at the beginning of each curve. Cyt2Aa2 of 25 µg/mL (1 µM) was filled into QCM-D chamber; then, the flow was paused in order to evaluate the lipid binding of Cyt2Aa2 protein for 2 h. After that, the systems were rinsed by PBS, as indicated by the arrows. The solid and dash arrows indicate points of protein injection and buffer rinsing, respectively. (**A**) The quantitative lipid binding of Cyt2Aa2 protein is observed in decreasing of ∆F. (**B**) The viscoelastic property of the layer is coincidently determined with increasing of ∆D. (**C**,**D**) Zoom-in of ∆F plot and ∆D plot for the first 60 min, respectively. These plots show the evolution of protein–lipid binding at the early time of incubation before reaching the saturated state.

Once the lipid bilayers were successfully formed, Cyt2Aa2 solution of 25 µg/mL (1 µM) was introduced into the system and Cyt2Aa2-lipid binding was evaluated until it reached a saturated state (for 2 h) (Figure 2). At binding saturation state, the total changes in frequency (∆F) and dissipation (∆D) values were similar for 5:0, 5:0.2, 5:1, and 5:2 (POPC:Chol) ratios. However, the binding rate was different (Table 2). The corresponding values for the saturated point were about −30 Hz and 2.5 × 10^−6^ for the ∆F and ∆D, respectively. Exceptionally, the 5:3 (POPC:Chol) ratio presented different binding behavior. The ∆F decreased monotonically with time without reaching equilibrium after two hours (∆F ~(−207) Hz), while the ∆D increased steadily in the meantime, denoting a very viscous layer (∆D ~43 × 10^−6^). Figure 2C depicts the adsorption kinetics (slope of ∆F curve) of all POPC:Chol ratios for the first 60 min. To evaluate the rate of protein binding, the ∆F plots (indicating mass adsorption) were fitted with a single exponential decay equation: $F_{t}=F_{0}+Ae^{-t/\Gamma }$, where *A* is the amplitude, *t* is the experimental time, and *Γ* is the time constant of decay (Figure S3). The binding constant is determined by *Γ*; shorter times indicate faster protein–lipid binding and vice versa (Table 2). The binding rate of Cyt2Aa2 protein was faster for the lipid bilayer containing higher cholesterol content, 5:1 and 5:2 (POPC:Chol), in which *Γ* = 3.2 and 2.1 min, respectively. Although the fastest adsorption occurred for the 5:3 (POPC:Chol) ratio (the slope of decay is the steepest), the *Γ* value could not be determined, since no saturation was achieved.

**Figure 3:**
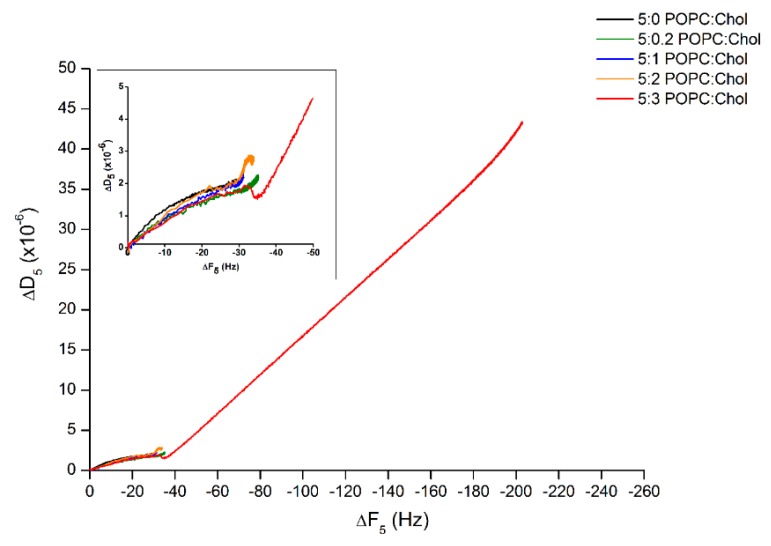
Analysis of the ∆D–∆F plot of Cyt2Aa2 protein binding on the lipid bilayers with different levels of cholesterol content. The frequency and dissipation values of Figure 2 were plotted together in order to correlate the deposited mass and viscosity at each time point of protein binding. The behavior of protein–lipid binding was considered according to the direction of plots. Inlet depicts the blow up of the behavior of lipid binding until reaching the saturated state.

Furthermore, to determine the behavior of lipid binding, ∆D was plotted against ∆F (∆D–∆F plot). Figure 3 reveals that the processes of protein–lipid binding were mostly similar for the first 25 min even if the binding rates were different. Interestingly, after 25 min of incubation, Cyt2Aa2 protein continuously bound on the bilayer with highest cholesterol content, 5:3 (POPC:Chol) ratio. Specifically, the ∆D–∆F plot shows a slight decrease before the growth of ∆F and ∆D became linearly proportional, which possibly indicates an initial protein–lipid structure arrangement. Subsequently, further Cyt2Aa2 protein adsorption delivered a more dissipating hybrid layer (generating a fluid-like protein–lipid layer). 

**Figure 4:**
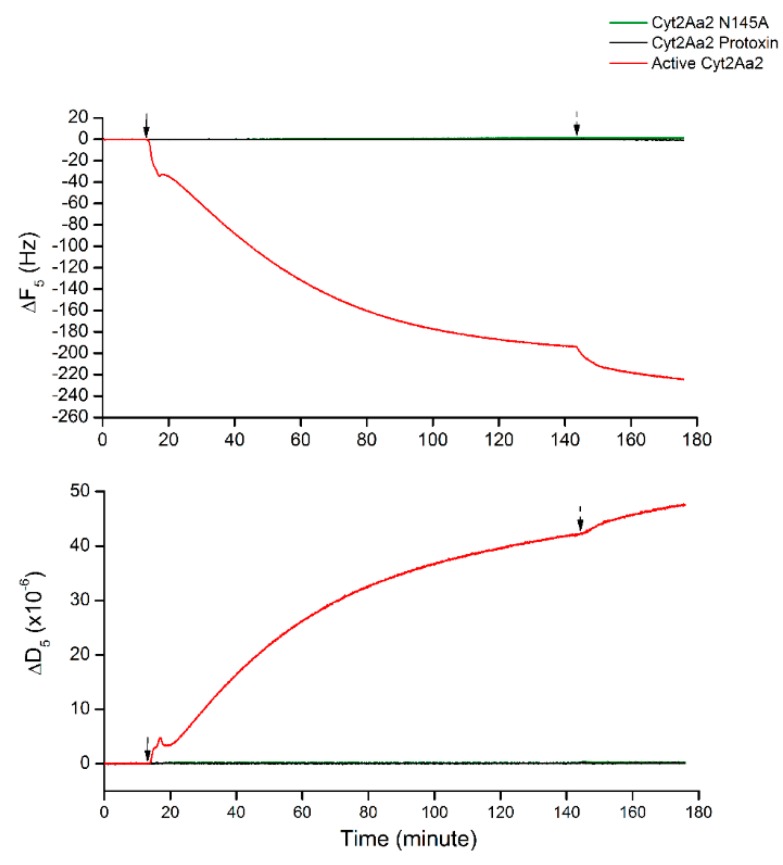
Negative protein binding of protease-inactivated Cyt2Aa2 and inactive Cyt2Aa2 N145A on the lipid bilayer of 5:3 (POPC:Chol) ratio. All protein solutions of 25 µg/mL were filled into QCM-D chambers, then the solution flow was paused for 2 h. The protein–lipid binding was considered by ∆F and ∆D changing. The solid and dash arrows indicate to points of protein injection and buffer rinsing, respectively.

Since Cyt2Aa2 protein binding behavior was changed at a large cholesterol content in lipid bilayer (5:3 POPC:Chol), the non-specific binding was characterized. The interactions of such bilayer with both protease-inactivated Cyt2Aa2 wild type and Cyt2Aa2 N145A (inactive mutant) [22] were investigated as a negative control. Figure 4 shows that neither inactivated Cyt2Aa2 nor Cyt2Aa2 N145 bind on the lipid bilayer, which neither changed in ∆F or in ∆D with time. These results confirmed that the change of binding behavior of Cyt2Aa2 protein on lipid bilayer with highest content cholesterol involved specific binding.

### 2.3. Nanostructure of the Cyt2Aa2/Lipid Bilayer

**Figure 5:**
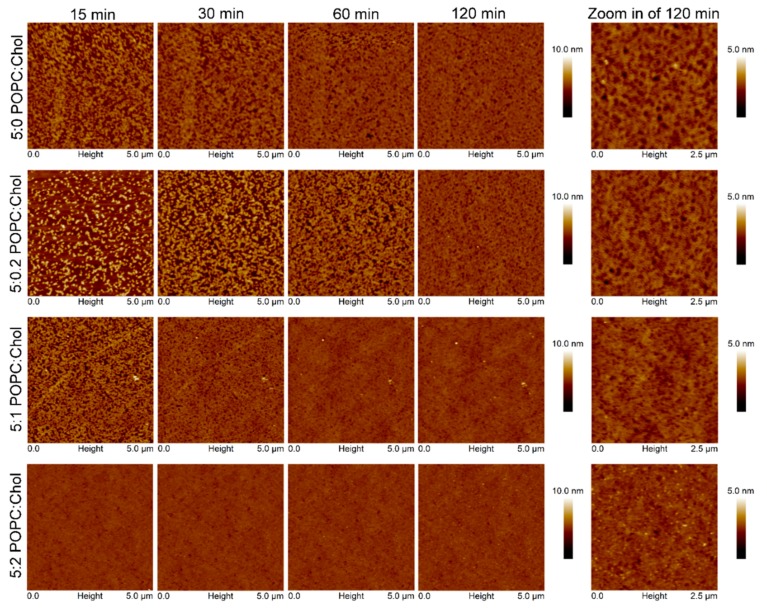
Time sequence AFM images of Cyt2Aa2 protein binding on lipid bilayers with different levels of cholesterol content. Once the lipid bilayer formation was confirmed by tip indentation, Cyt2Aa2 of 25 µg/mL was introduced into the fluid cell chamber. AFM images were collected in tapping mode with scan rate of 1–2 Hz in 5 µm × 5 µm. The final topographic images are zoomed in to reveal the topographic pattern of protein–lipid binding.

**Figure 6:**
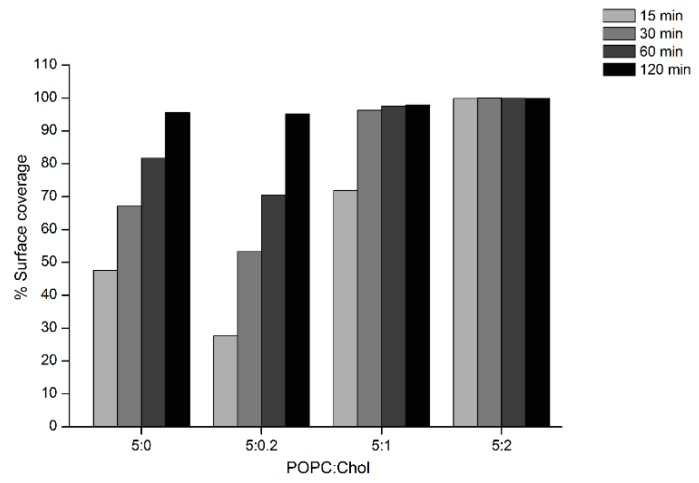
Analysis of AFM topographic image of Cyt2Aa2 protein binding on lipid bilayers. The surface coverage of protein binding as a function of time for different lipid/Chol bilayers was analyzed from set of image of Figure 5 (5 µm × 5 µm). The increasing of % surface coverage refers to the quantitative lipid binding. The AFM images were analyzed with bearing analysis of the NanoScope analysis program.

AFM experiments were carried out to investigate the surface topography and properties of the Cyt2Aa2/lipid bilayer. In these experiments, Cyt2Aa2 (25 µg/mL) was exposed to different ratios of POPC/Chol bilayers: 5:0, 5:0.2, 5:1, 5:2, and 5:3. Once the lipid bilayers were formed, active Cyt2Aa2 solution was introduced in the liquid chamber and the Cyt2Aa2/lipid topography was imaged as a function of time. After protein injection, the AFM-cantilever needed to equilibrate due to thermal drift, and the early stage of Cyt2Aa2 protein binding could not be detected. However, after 15 min of incubation a difference in protein binding was detected. At this point, the surface coverage increased along with the amount of cholesterol in lipid bilayer; partial coverage was observed for 5:0 and 5:0.2 (POPC:Chol) ratios, larger coverage was seen for 5:1 (POPC:Chol) ratio, and the full coverage was detected for 5:2 (POPC:Chol) ratio (Figure 5). A detailed analysis of the surface coverage versus time is depicted in Figure 6. At 5:0 and 5:0.2 (POPC:Chol) ratios, the surface coverage increases in a linear fashion with time, reaching a maximum value after two hours. For the 5:1 (POPC:Chol) ratio, full coverage is achieved after 30 min. Finally, full coverage at 15 min takes place for the 5:2 mixture, being the value of the surface coverage of the layer constant after this time. Particularly interesting was the evolution of the surface properties with time when CytAa2 protein was exposed to POPC/Chol bilayer of 5:0 and 5:0.2 ratios. Between the first protein aggregation steps and full coverage after two hours, the formation of holes could be observed. A height profile analysis revealed hole depths were about 1.5–3.0 nm, and the hole percentage within the layer was ca. 5% of the total area (Figure S4). The analysis of the surface topography for the 5:0, 5:0.2, 5:1, and 5:2 (POPC:Chol) ratios after two hours indicated that the number of holes was reduced when the amount of cholesterol was increased in the bilayer (Figure 5). 

**Figure 7:**
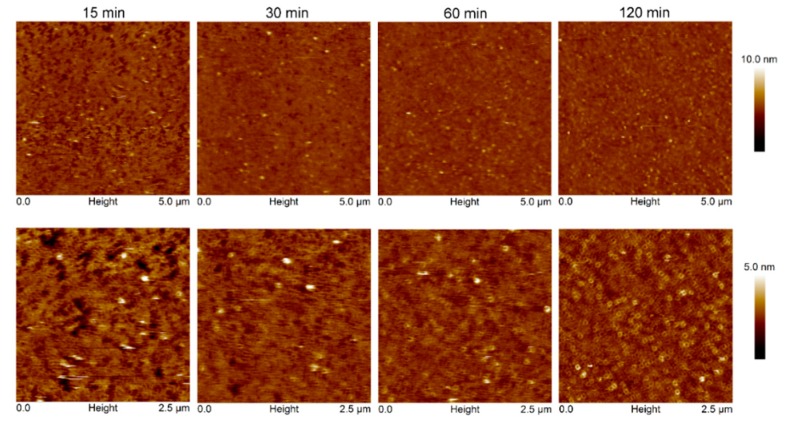
Time sequence AFM images of Cyt2Aa2 protein binding on the lipid bilayer of 5:3 (POPC:Chol) ratio. Once the lipid bilayer formation was confirmed by tip indentation, Cyt2Aa2 protein of 25 µg/mL was introduced into the fluid cell chamber. AFM images were collected in tapping mode with scan rate of 1–2 Hz in 5 µm × 5 µm. The upper panels are the full scan size images and the lower panels are zoom-in images.

**Figure 8:**
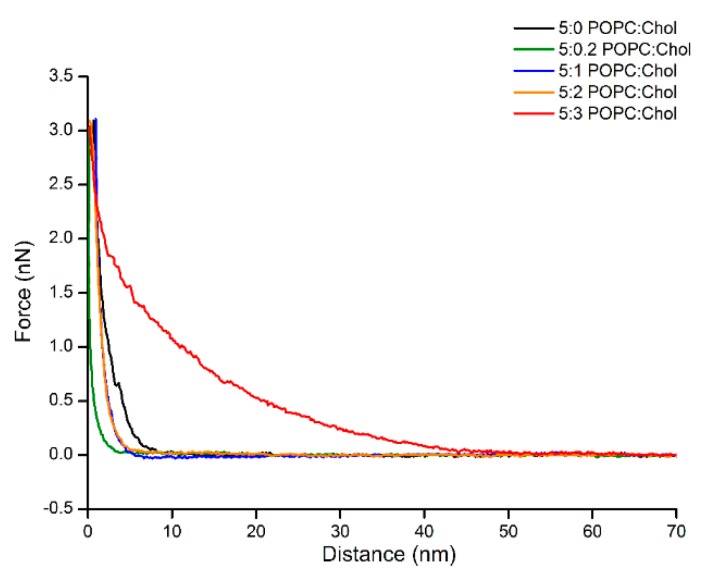
Approaching force–distance curves of Cyt2Aa2/lipid layers with different levels of cholesterol content. The AFM tip was moved close to the surface until reaching the contact point (close to zero point), then force was increased to the set point (3 nN). The force–distance curve was carried out at 1 Hz. Long-range repulsion is observed in the case of protein adsorption on 5:3 (POPC:Chol) ratio.

Interestingly, the adsorption of Cyt2Aa2 protein on the bilayer of 5:3 (POPC:Chol) ratio delivered different surface properties for the Cyt2Aa2/lipid layer at early stage. After 15 min, strip patterns and holes were observed on the surface (Figure S5). Subsequently, the Cyt2Aa2-lipid layer rearranged after about 30 min. The topography images indicated that the strip pattern disappeared, while a ring shape form appeared. After two hours, ring shapes were the main pattern observed on the hybrid layer surface (Figure 7). Furthermore, force–distance curves were carried out on all the samples at two hours (Figure 8). The results showed a strong repulsion between the AFM tip and Cyt2Aa2/lipid layer for the 5:3 (POPC:Chol) ratio system. The AFM-tip experienced a repulsive force at about 40 nm, possibly a long-range electrostatic repulsion, due to the charge of the adsorbed protein on the POPC/Chol bilayer. The force–distance curves were very similar for the other four systems exhibiting negligible repulsion. AFM results might indicate a change in binding behavior of Cyt2Aa2 protein on the bilayer of 5:3 (POPC:Chol) ratio, as shown by QCM-D. Two structures were found from the surface topography studies: a strip pattern with holes, and ring shapes (which might refer to two binding steps of ∆D–∆F plot).

## 3. Discussion

### 3.1. The Role of Cholesterol on Lipid Bilayer 

The characterization of the liposomes (in bulk) showed that the liposome size was enlarged upon increasing of cholesterol content in lipid membrane and the zeta potential value remained similar because of the zwitterionic nature of choline head group. These findings are in agreement with the results concerning similar lipid systems [23]. In addition, cholesterol content also affected lipid bilayer formation via liposome rupture. It was observed that for the lipid bilayer of pure POPC and the 5:0.2 (POPC:Chol) ratio, liposomes were spontaneously formed (Figure S1). In turn, the variation of the ∆F versus time for higher POPC:Chol ratios showed a second decrease in ∆F (approx. at 30 min), which could indicate the formation of liposomes inserted in the lipid bilayer leading to bilayer defects. Therefore, complete lipid bilayers were formed by buffer rinsing when the second decreasing of ∆F was detected. The additional step of decreasing in ∆F might be explained by interactions between the recent formed bilayer and liposomes in bulk solution during cholesterol flip-flop across lipid bilayers [24,25,26]. Hence, the buffer flow is likely to remove the excess of intact liposomes and weak adsorbed liposomes from the bulk and the surface. A similar result was found in the study of lipid bilayer formation of liposome containing phase separation induced by cholesterol [27]. According to the POPC-Chol phase diagram [28], the POPC:Chol mixtures used in this work present different lipid phases at 25 °C: a liquid disordered phase (*l_d_*) corresponds to 5:0 and 5:0.2 (POPC:Chol) ratios, a coexistence liquid disordered-liquid ordered phase (*l_o_*–*l_d_*) can be attributed to 5:1 ratio, while a liquid ordered phase (*l_o_*) is related to the 5:2 and 5:3 (POPC:Chol) ratios. Here, cholesterol might be involved in the lipid bilayer formation process via affecting the lipid bilayer packing related to the lipid phase state.

Furthermore, AFM experiments were carried out in parallel to characterize the POPC/Chol bilayer. Height and phase measurements indicate that all resulting of POPC/Chol bilayers are a flat surface (Figure S2). Moreover, force–distance curves indicated that the POPC/Chol bilayers were slightly thicker upon the increasing of cholesterol content, ranging from 4.0 to 5.5 nm (increasing approximately 1.0 nm) (Figure 1). These values are comparable to the thickness of supported lipid bilayers previously determined by AFM [29,30]. As previous studies state, the interplay between cholesterol and phospholipid molecules promotes lateral ordering of lipid acyl chain leading to a lipid phase change from *l_d_* phase to *l_o_* phase [31,32,33]. Consequently, it can be concluded that cholesterol can enlarge liposome size and increase lipid bilayer thickness.

### 3.2. Protein Binding as a Function of Cholesterol Content in Lipid Bilayer

The binding of Cyt2Aa2 protein on lipid bilayers was similar for the following POPC:Chol ratios: 5:0, 5:0.2, 5:1, and 5:2 (Figure 2). The ∆F and ∆D at the saturated state were about −30 Hz and 2.5 × 10^−6^, respectively. In addition, ∆D–∆F plots also indicated similar lipid binding behavior among them (Figure 3). The lipid binding rate increased relative to the amount of cholesterol in lipid bilayer by time constant decay (*Γ*) (see values in Table 2). Thus, cholesterol in our case speeds up the binding process (binding also occurred when no cholesterol was present in the bilayer). The presence of cholesterol in lipid bilayer might promote Cyt2Aa2 binding by reducing lipid fluidity (less lipid mobile) that increases a chance for Cyt2Aa2 protein docking on lipid molecules. Unfortunately, electrophoretic measurements did not provide large differences in the zeta potential value as a function of cholesterol content. Interestingly, Cyt2Aa2 protein–lipid binding changed significantly for the lipid mixture of 5:3 (POPC:Chol) ratio. At this lipid mixture, the ∆F, ∆D, and ∆D–∆F curves (indicating binding behavior) were different from the other mixtures. The ∆F and ∆D could not reach a plateau after two hours of incubation; their values were ∆F ~(−207) Hz and ∆D ~43 × 10^−6^, respectively. In particular, the large dissipation value indicated a more viscous layer, suggesting that high cholesterol content in the lipid bilayer may lead to a new assembled Cyt2Aa2-lipid structure. The high dissipation values of Cyt2Aa2 could be indicative of beta-pore formation; a similar effect was observed for α-hemolysin during pore formation in lipid bilayers [34]. The analysis of ∆D–∆F plot revealed the binding behavior of Cyt2Aa2 protein on the lipid bilayer with the highest cholesterol content could consist of two processes. The first step process was similar to the other four lipid mixtures but the speed of protein adsorption was faster (∆F slope is steeper). Thus, the first protein–lipid layer arrangement led to a more rigid layer (decreasing of ∆D), while in a second step further changes in ∆F and ∆D values took place (Figure 3). The new structure after rearrangement might provide a place for further molecule binding, e.g., Cyt2Aa2 protein, water, and ions. 

Moreover, AFM time sequence imaging and force curve measurements provided additional information of lipid binding and surface property. The increase of lipid binding rate upon higher cholesterol content revealed by surface coverage of AFM image analysis (Figure 6) agrees with the result of QCM-D indicating by *Γ* value. Although the change in ∆D of QCM-D results indicated that the viscosity of the final structures was similar (at two hours), AFM measurements revealed differences in surface topography. A strip pattern with holes was observed for 5:0 and 5:0.2 (POPC:Chol) ratios, whereas no pattern could be seen for 5:1 and 5:2 (POPC:Chol) mixtures. The strip pattern of Cyt2Aa2 looks similar to a filament-like oligomer of volvatoxin 2 (homology protein structure) in solution [35]. 

On the contrary, the surface structural change as a function of time of the Cyt2Aa2-lipid layer for the 5:3 (POPC:Chol) ratio corresponded with QCM-D results. At 15 min of incubation, the interaction of Cyt2Aa2 with the 5:3 lipid/Chol layer was observed, leading to a strip pattern structure as found for 5:0 and 5:0.2 (POPC:Chol) ratios. This result agrees with the ∆D–∆F plot that indicates a similar protein–lipid interaction. Further incubation (for 2 h) showed the evolution of the surface properties of Cyt2Aa2-lipid hybrid layer of 5:3 (POPC:Chol), while the other lipid/Chol ratios did not experience any change. QCM-D and force–distance curves confirmed that the hybrid layer of the 5:3 (POPC:Chol) ratio involved more compliance, showing a different surface charge distribution (repulsion interaction between tip and layer). The diversity in the surface topographic pattern might be due to different in protein–protein, lipid–lipid, and protein–lipid interactions influenced by the cholesterol content in the lipid bilayers.

Finally, we assume that these results are related to the biological activity of Cyt2Aa2 protein. Cyt2Aa2 protein is toxic to mosquito larvae and also exerts cytolytic activity against red blood cells [22]. Both cell membranes contain different lipid:Chol composition. As the results show, Cyt2Aa2 protein has distinct lipid binding behavior on 5:0.2 and 5:3 (POPC:Chol) bilayers, which mimics the mosquito cell membrane [20] and the red blood cell membrane [17,21], respectively. The results suggest that Cyt2Aa2 protein may interact and change the structure of the cell membrane of both cell types in different ways. 

## 4. Materials and Methods 

### 4.1. Reagents and Buffer 

1-palmitoyl,2-oleoyl-snglycero-3-phosphocholine (POPC) and cholesterol (Chol) were purchased from Sigma-Aldrich, Germany. The lipids were dissolved in chloroform and aliquot. Then, the organic solvent was evaporated under nitrogen stream and kept at −20 °C. Phosphate-buffered saline (PBS) pH 7.4 was prepared from PBS tablet (Sigma-Aldrich, Darmstadt, Germany). The buffer tablet was dissolved in ultra-pure water (Milli-Q water, Merck, Darmstadt, Germany) and then filtrated through 0.22 µm filter (Whatman, GE Health care Life science, Chicago, IL, USA). 

### 4.2. Protein Preparation

Cyt2Aa2 protein wild type and mutant Cyt2Aa2 N145A were prepared as described previously by B. Promdonkoy [36]. To obtain active protein, Cyt2Aa2 protein was solubilized in alkaline condition (50 mM carbonate buffer, pH 10.0) at 30 °C for 1 h. Soluble Cyt2Aa2 was separated by centrifugation at 10,000× *g* for 10 min at 25 °C. Subsequently, Cyt2Aa2 protein was activated by 2% (*w*/*w*) chymotrypsin (Sigma-Aldrich, Germany) at 30 °C for 2 h. Protein purity and molecular weight of Cyt2Aa2 were evaluated by SDS-PAGE (Invitrogen, Waltham, MA, USA), and protein concentration was determined by UV 280 nm absorption (Hitachi U2900, Tokyo, Japan).

### 4.3. Liposome Preparation and Characterization

The lipid compositions to prepare liposomes were POPC and cholesterol. The liposomes were prepared as described previously [37]. In brief, POPC and cholesterol were mixed with different weight ratios to 5:0.2, 5:1, 5:2, and 5:3 (POPC:Chol). After that, organic solvent was evaporated under a gentle nitrogen stream to form a lipid film. The residual solvent was removed by keeping the lipid film under nitrogen stream for 1 h. The lipid film was hydrated with PBS at ambient temperature (25 °C) for at least 1 h. The hydrated film was intermittently vortexed during incubation until complete suspension. The liposome mixture was repeatedly pressed through 50 nm-polycarbonate membrane 21 times by using a mini-extruder (Avanti, Alabaster, AL, USA) and then stored at 4 °C. 

The liposome size and zeta potential were determined with a Zetasizer Nano ZS (Malvern Instrument, Worcestershire, UK). The liposome stocks were diluted to concentration of 50 µg/mL in order to measure the liposome size by dynamic light scattering. For the zeta potential, the liposomes were measured by laser doppler microelectrophoresis: 100 μL of 1 mg/mL liposome solution was loaded into a bottom of folded capillary cell (DTS1070, Malvern Instrument, UK) containing PBS. The liposomes were left at ambient temperature for 15 min before measurement. All of measurement were carried out at 21 °C. Each of samples was measured five times from two independent batches of liposome (10 measurements).

### 4.4. Quartz Crystal Microbalance with Dissipation (QCM-D) Measurement

Silicon-coated quartz sensors (QSX 303, Biolin Scientific, Gothenburg, Sweden) were used for all of QCM-D measurements. The sensors were sequentially cleaned with sonication in 2% (*w*/*w*) SDS solution for 15 min, ultra-pure water for 10 min, and ethanol for 10 min. Then, the sensors were dried under nitrogen stream. The organic residues on the sensor surface were removed concomitant with surface oxidized in UV/Ozone chamber (Bioforce Nanosciences, Salt Lake City, UT, USA) for 30 min. The sensors were mounted into the QCM-D chamber as soon as possible to avoid any contamination.

The QCM-D experiments were carried out with Q-Sense E4 QCM-D (Biolin Scientific, Sweden). The fundamental frequency and the frequencies of the overtones (3rd, 5th, 7th, 9th, 11th, and 13th) were evaluated prior the experiment running. The changes of frequency (∆f) and dissipation (∆D) values of the 5th overtone are presented for all experiments. The crystal sensors were incubated with PBS (pH 7.4) under flow condition with a flow rate of 50 μL/min until reaching to a stable baseline (at least 1 h). The lipid bilayer formation on top of sensor surface was described previously [23]. Briefly, 0.1 mg/mL liposome solutions were slowly filled into the QCM-D chamber with flow rate of 50 μL/min. Once the signal of lipid bilayer formation was observed, the excess liposomes were removed by buffer rinsing until a stable signal was obtained. The protein solution of Cyt2Aa2 was introduced into the system at flow rate of 50 μL/min. Then, the flow was stopped for 2 h in order to evaluate the protein–lipid binding process. The still unbound protein was flushed from the chamber with PBS at flow rate of 50 μL/min for 30 min. The experiments (at least three replications) were carried out at 25 °C. 

### 4.5. Atomic Force Microscope (AFM) Imaging

Lipid bilayers were formed on 1 cm x 1 cm silicon wafers (IMEC, Leuven, Belgium) by mean of liposome fusion. The silicon wafers were cleaned in a similar way to QCM-D sensor; sonication in 2% (*w*/*w*) SDS for 15 min, rinsing with ultrapure water and ethanol, and drying under nitrogen stream. Finally, the organic contaminates were removed concomitant with surface activation by plasma cleaner (Diener electronic, Ebhausen, Germany). The AFM probe and the silicon wafers were mounted into a closed liquid chamber. The solutions were exchanged by syringe injection. After introducing the solutions, the system was left to equilibrate until the deflection signal was stable. To form the lipid bilayer, the liposome solutions at 0.1 mg/mL were incubated over the silicon surface for 15 min and then the excess liposomes were flushed from the chamber. Once lipid bilayer formation was confirmed by means of tip indentation, the Cyt2Aa2 solution was added to the chamber. Cyt2Aa2-lipid binding was imaged as a function of time. The AFM images were obtained in tapping mode with DNP-S10 AFM cantilevers of nominal spring constant of 0.24 N/m (Bruker, Billerica, MA, USA). All experiments were carried outed with a JV-scanner controlled by NanoScope V controller (Bruker, USA) at a scan rate of 1–2 Hz at room temperature (~25 °C). Force–distance curves were performed at 1 Hz. The applied force on the sample was kept as low as possible to avoid damaging. The images were processed and analyzed with NanoScope Analysis program (Bruker, USA).

## 5. Conclusions

In this work, the cholesterol content was varied in the lipid bilayers in order to mimic the cell membrane of insect and mammalian cells. The results have shown that the cholesterol content (of a lipid bilayer) influences the protein–lipid binding of Cyt2Aa2 protein by increasing its binding rate and changing the surface properties of the final protein:lipid:Chol layer. However, the experimental results also show that in absence of cholesterol, Cyt2Aa2 protein also binds to the lipid bilayer. Furthermore, Cyt2Aa2 protein changes its binding behavior for the 5:3 (POPC:Chol) bilayer, which corresponds to the mammalian cell model. Our findings imply that the amount of cholesterol in the lipid bilayer affects the protein–lipid binding rate and the final structure of the Cyt2Aa2/lipid layer. In future work, supporting lipid bilayers made of different phospholipid compositions will be used to get more insight about the Cyt2Aa2-lipid bilayer interaction. This study leaves open questions related to the energetics and molecular mechanism of binding. Therefore, QCM-D, AFM, calorimetry, and electrochemistry experiments with Cyt2Aa2 mutants will be carried out to address these issues. Especially interesting will be the binding and the final nanostructure of the hybrid protein–lipid bilayer dependence on (amino acid) point mutations.

## Figures and Tables

**Table 1:** Final ∆F and ∆D values for the supported lipid bilayers formed with different levels of cholesterol content in the lipid membrane.

POPC:Chol Ratio (by Weight)	∆F_5_ (Hz)	∆D_5_ (×10^−6^)
5:0	−26.1 ± 1.2	1.6 ± 0.9
5:0.2	−29.6 ± 2.6	1.6 ± 0.6
5:1	−30.3 ± 0.5	1.9 ± 0.1
5:2	−37.1 ± 1.0	3.6 ± 0.5
5:3	−31.3 ± 0.6	2.2 ± 0.1

The values are showed in mean ± SD.

**Table 2:** Summary of the final change in frequency (∆F), dissipation (∆D), and time constant of decay (*Γ*) at saturated state of Cyt2Aa2 protein binding on lipid bilayers with different levels of cholesterol content.

POPC:Chol Ratio (by Weight)	∆F_5_ (Hz)	∆D_5_ (×10^−6^)	*Γ* (min)
5:0	−29 ± 2	2.2 ± 0.7	6.9
5:0.2	−33 ± 2	2.2 ± 0.3	6.3
5:1	−30 ± 1	2.7 ± 0.3	3.2
5:2	−32 ± 1	2.5 ± 0.9	2.1
5:3	−207 ± 4	43.7 ± 1.1	N/A

*Γ* of 5:3 (POPC:Chol) ratio cannot be determined, because the frequency value did not reach a saturated state. The values are written as mean ± SD.

## Supplementary Materials

The Supplementary Materials are available online at http://www.mdpi.com/1422-0067/19/12/3819/s1.

## Abbreviations

*Bt*	*Bacillus thuringiensis*
Cyt protein	Cytolytic protein
Cyt2Aa2 protein	Cytolytic protein from *Bacillus thuringiensis* subsp. *darmstadiensis*
QCM-D	Quartz crystal microbalance with dissipation
AFM	Atomic force microscope
POPC	1-palmitoyl,2-oleoyl-snglycero-3-phosphocholine
Chol	cholesterol
