# Supplementary material for: Cholesterol Increases Lipid Binding Rate and Changes Binding Behavior of Bacillus thuringiensis Cytolytic Protein
Source: Int J Mol Sci. 2018 Nov 30;19(12):3819. doi: 10.3390/ijms19123819 (PMC6321300; doi:10.3390/ijms19123819)
Supplement: Supplementary file 1 [file ijms-19-03819-s001.pdf]

Table S1. Summary of the properties of the liposomes formed with a different cholesterol content in the lipid membrane.

POPC:Chol ratio (by weight)	Liposome	
	size (nm)	ζ (mV)
5:0	110.8 ± 7.6	-3.4 ± 3.0
5:0.2	107.9 ± 8.7	-4.0 ± 2.7
5:1	119.0 ± 1.9	-4.4 ± 1.7
5:2	133.8 ± 2.2	-3.2 ± 1.1
5:3	137.5 ± 6.2	-3.8 ± 1.0

Note. The values were presented in mean ± SD.

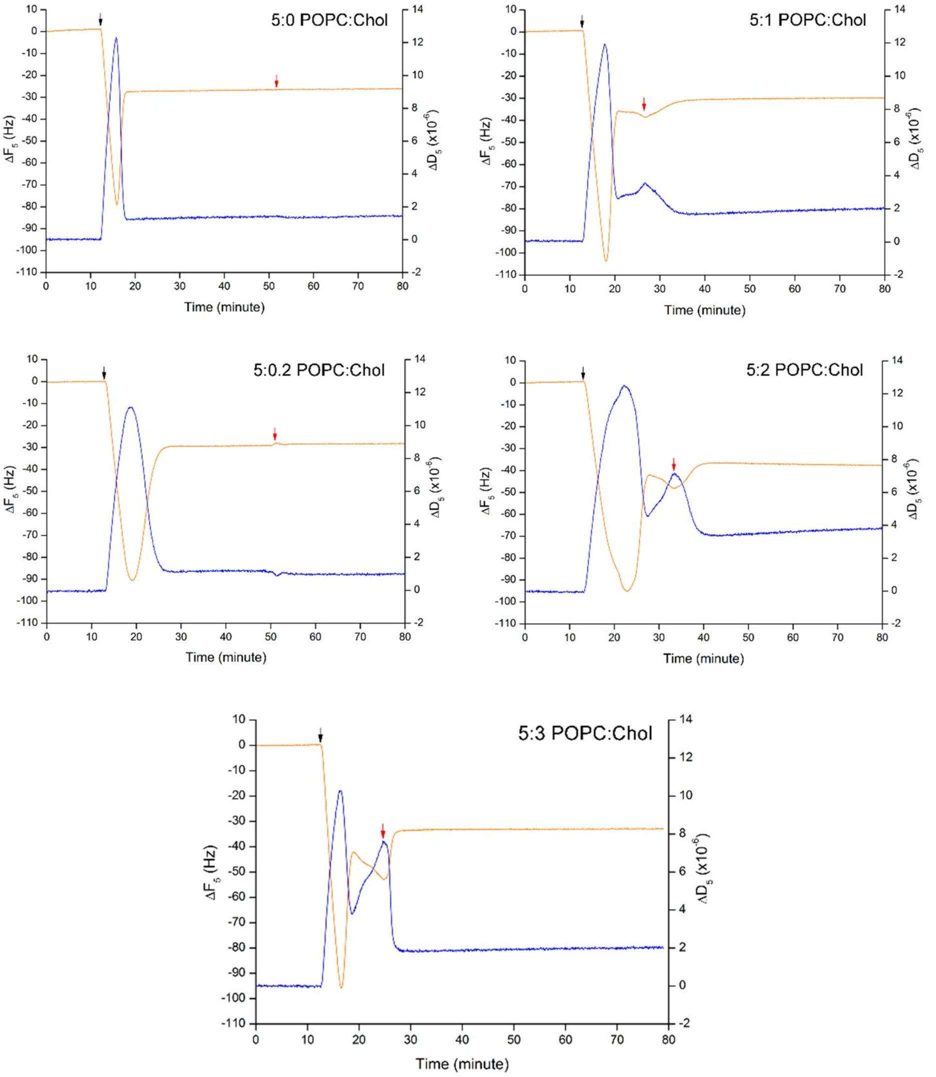

Figure S1. Lipid bilayer formation of liposome containing different cholesterol content. Lipid bilayers were formed by liposome fusion method. Liposome solutions of 0.1 mg/ml were filled into QCM-D chamber at flow rate of 50 μl/min until the ΔF and ΔD values indicated liposome rupture. The excess of liposomes was flushed from the chamber until a stable base line was observed. The black and red arrows indicate to liposome injection and buffer rinsing, respectively. The frequency plot is indicated in orange color whereas the dissipation plot is indicated in blue.

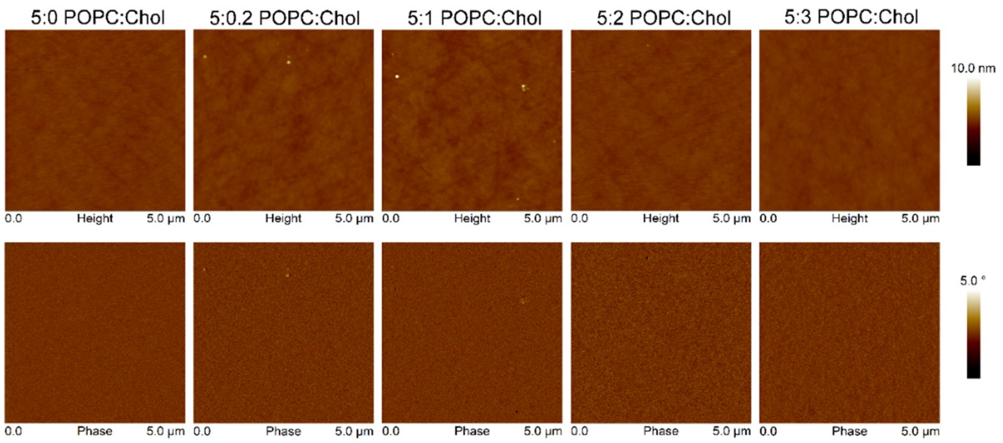

Figure S2. AFM topographic images of lipid bilayer containing different cholesterol content. The lipid bilayers were formed by liposome fusion method. Liposome solutions of 0.1 mg/ml were introduced into the fluid cell and incubated over the silicon surface for at least 15 minutes. Then, the excess of liposomes was flushed from the system. The existence of lipid bilayers on the surface was evaluated by AFM tip indentation.

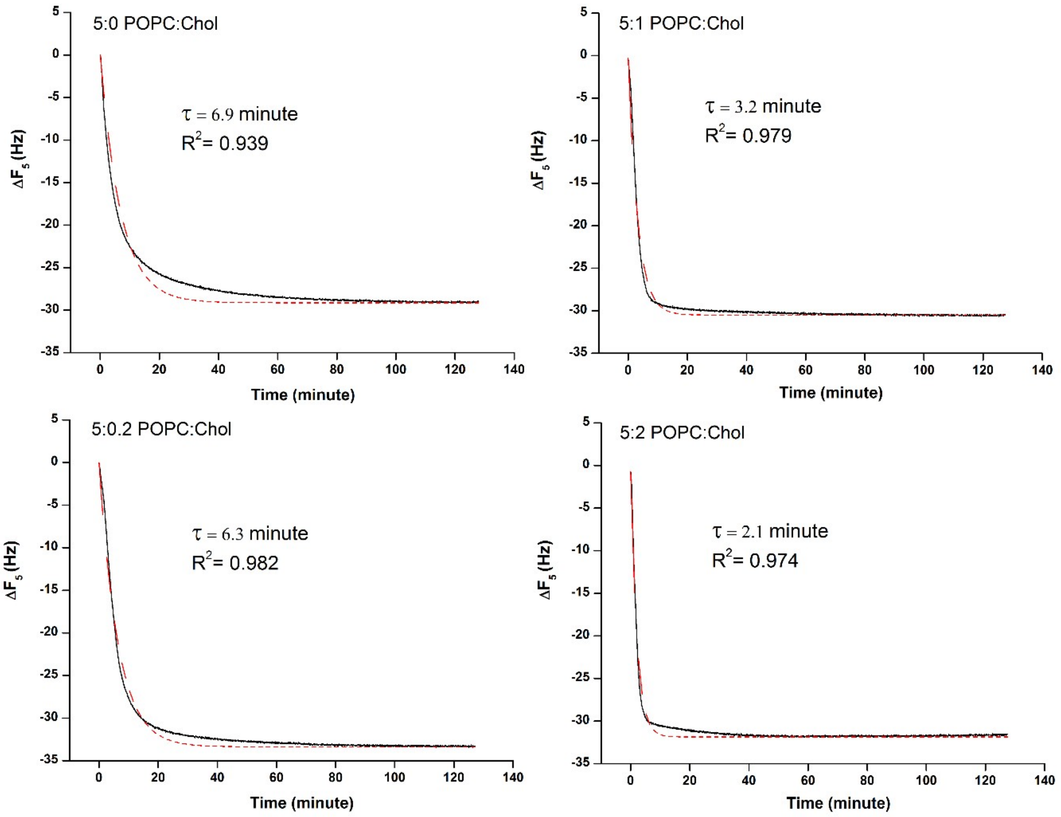

Figure S3. Determination of lipid binding rate by curve fitting. Each ΔF curve of protein-lipid binding of lipid bilayer with different cholesterol content is fitted with single exponential decay equation: $F_t = F_0 + Ae^{-t/\Gamma}$ where A is the amplitude, t is the experimental time, and Γ is the time constant of decay. Experimental data are plotted as a solid black line whereas the equation model is plotted as a dash red line. Γ refers to the rate of protein-lipid binding; a faster binding is indicated by low number and vice versa.

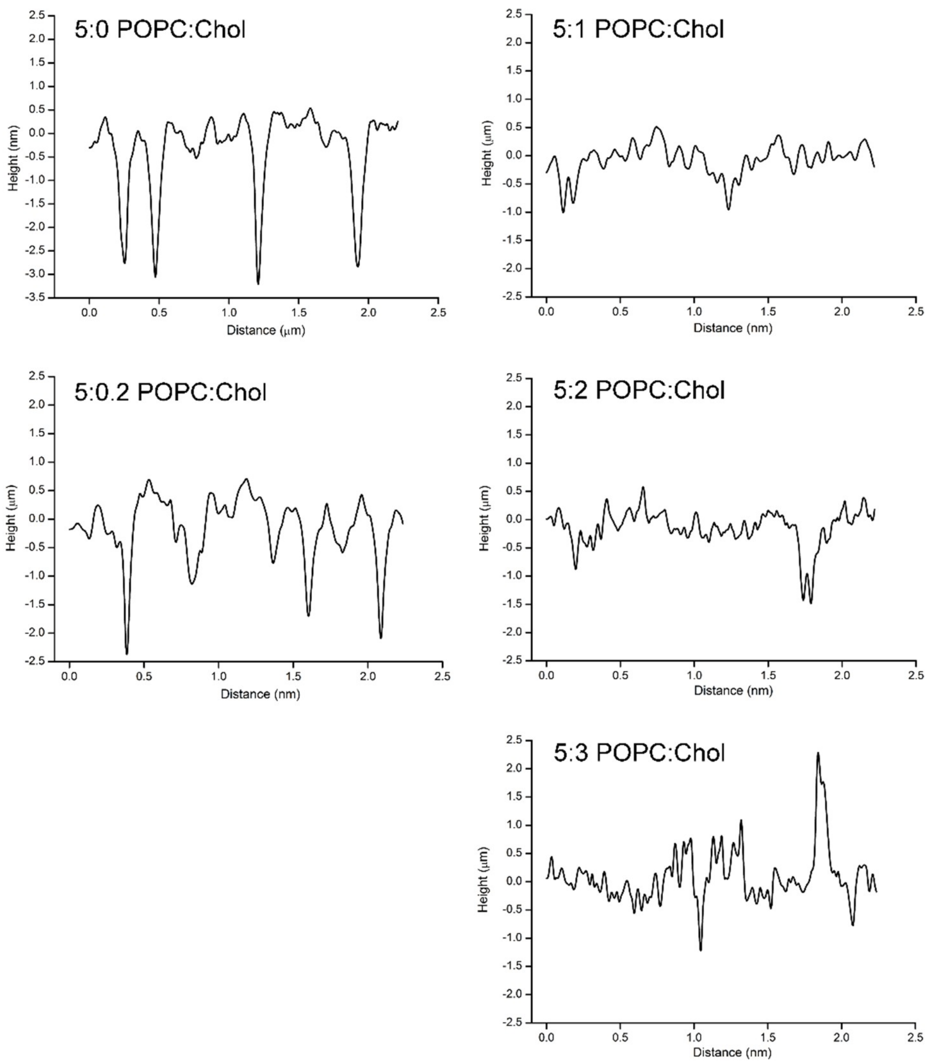

Figure S4. Height profile analysis of Cyt2Aa2/lipid layers with different cholesterol content after 2 hours of incubation. The hole depth and thickness of the hybrid layer were determined by cross section analysis of the original AFM height images. Note that small cholesterol content lead to a larger difference in height profile.

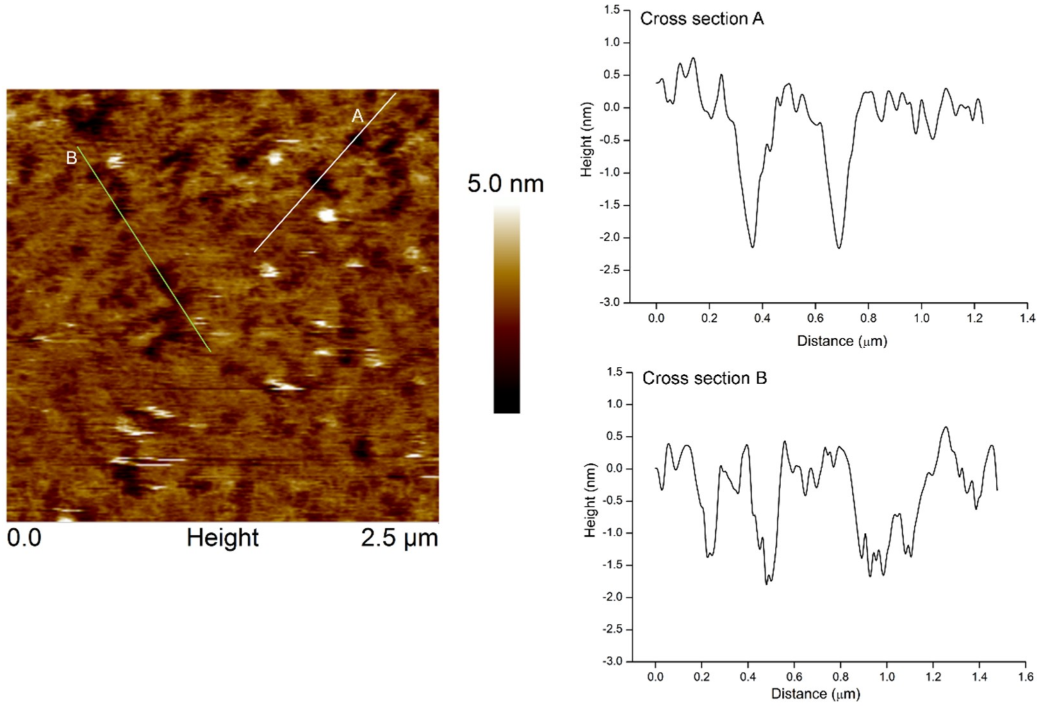

Figure S5. Height profile analysis of Cyt2Aa2/lipid layers of 5:3 (POPC:Chol) at 15 minutes of incubation. The analysis of the AFM height images permits to determine the (minimum) depth of the holes, which is about 2 nm. Note that the "V-shape" is given by the geometry of the tip.
